# Synergy and antagonism in regulation of recombinant human INO80 chromatin remodeling complex

**DOI:** 10.1093/nar/gkw509

**Published:** 2016-06-02

**Authors:** Oliver Willhoft, Rohan Bythell-Douglas, Elizabeth A. McCormack, Dale B. Wigley

**Affiliations:** Section of Structural Biology, Department of Medicine, Imperial College London, London SW7 2AZ, UK

## Abstract

We have purified a minimal core human Ino80 complex from recombinant protein expressed in insect cells. The complex comprises one subunit each of an N-terminally truncated Ino80, actin, Arp4, Arp5, Arp8, Ies2 and Ies6, together with a single heterohexamer of the Tip49a and Tip49b proteins. This core complex has nucleosome sliding activity that is similar to that of endogenous human and yeast Ino80 complexes and is also inhibited by inositol hexaphosphate (IP_6_). We show that IP_6_ is a non-competitive inhibitor that acts by blocking the stimulatory effect of nucleosomes on the ATPase activity. The IP_6_ binding site is located within the C-terminal region of the Ino80 subunit. We have also prepared complexes lacking combinations of Ies2 and Arp5/Ies6 subunits that reveal regulation imposed by each of them individually and synergistically that couples ATP hydrolysis to nucleosome sliding. This coupling between Ies2 and Arp5/Ies6 can be overcome in a bypass mutation of the Arp5 subunit that is active in the absence of Ies2. These studies reveal several underlying mechanisms for regulation of ATPase activity involving a complex interplay between these protein subunits and IP_6_ that in turn controls nucleosome sliding.

## INTRODUCTION

The compaction of DNA into chromatin in eukaryotes provides advantages for stability of the genetic material but also problems for processes such as repair of DNA damage. Consequently, systems have evolved to deal with DNA damage within the context of nucleosomes. Nucleosomes are remodeled by sliding them away from damage sites to allow access by the repair machinery. Such chromatin remodelers can be simple single subunit proteins such as Chd1 or more complex, multi-subunit systems such as RSC or Ino80 (1). Single subunit systems are able to slide nucleosomes so it is something of a mystery why such complicated, multi-subunit machines also exist that appear to perform similar functions.

The Ino80 family of remodelers contain a conserved core of subunits together with around half a dozen additional proteins that are species-specific (1). In addition to the main 180 kDa Ino80 remodeling subunit, that contains a double-stranded DNA translocase motor of the helicase Superfamily 2 (2), the conserved core proteins include nuclear actin and several members of the actin-related protein (ARP) family (Arp4, Arp5 and Arp8). Although details of the functions of these subunits in the remodeling reaction remain unclear, several have been shown to interact with histones or nucleosomes in solution (3,4). Ino80 complex also contains two subunits (Tip49a and Tip49b in humans, RvbL1 (or Rvb1) and RvbL2 (or Rvb2) in yeast) with homology to the bacterial RuvB hexameric helicase. The function of these subunits is also unclear. Finally, two additional proteins of unknown function (Ies2 and Ies6) complete the conserved core.

Crystal structures of several components of the Ino80 complex have been determined including the Tip49/RvbL (5–7), actin (8), Arp4 (9) and Arp8 (4,10) subunits. Electron microscopy studies of the yeast RvbL1/2 complex (11–13) have been controversial with the presence of both hexamers and dodecamers being reported. Electron microscopy studies of the intact Ino80 complex have also provided conflicting interpretations with some studies suggesting a single RvbL1/2 heterohexamer in the yeast complex (14,15) but others proposing a dodecamer (16). EM studies of the related yeast Swr1 complex, that contains about half of the same protein subunits as the Ino80 complex, have consistently shown a single RvbL1/2 heterohexamer (14,15).

Ino80 complex has been isolated from both yeast and humans (17,18) using tagged subunits and pulling down the associated proteins from endogenous cell extracts. Such studies have been able to demonstrate the basic biochemical properties of the complexes but have been limited by the quantities of protein that can be prepared by this method. In order to overcome some of these issues, we have prepared a recombinant minimal ‘core’ complex of human Ino80 in insect cells that comprises a truncated Ino80 subunit (shown previously to be active (19)) together with actin, Arp4, Arp5, Arp8, Ies2, Ies6 and the Tip49a/b complex. This complex has similar activity in nucleosome sliding assays to that reported for intact human and yeast complexes prepared from endogenous sources (15,17–20). The core complex contains a single Tip49a/b heterohexamer. Yeast Ino80 complex is inhibited by inositol hexaphosphate (IP_6_) (21,22). We show that the human Ino80 core complex (hIno80) is also inhibited by IP_6_ and determine that the basis of this inhibition is a reduction in nucleosome affinity. Consequently, the observed effect on ATPase activity is a result of a reduced stimulation by nucleosomes rather than inhibition *per se*. We also used our recombinant system to prepare complexes that individually lacked Ies2, Arp5/Ies6 and Ies2/Arp5/Ies6. We have characterized these complexes, shedding light on the roles of these subunits in regulating ATPase activity and coupling this to sliding activity in hIno80. Additionally, we show that requirement of the conserved Ies2 subunit for nucleosome sliding activity can be bypassed by a mutant Arp5 protein.

## MATERIALS AND METHODS

### Cloning and purification of human Ino80 complexes

Genes for a nine subunit human Ino80 core complex comprising a truncated form of the Ino80 (residues 267–1556 with an N-terminal 8-histidine and C-terminal twin Strep-tag® II (Ino80ΔN)). actin, Arp4, Arp5, Arp8, Ies2, Ies6, Tip49a and Tip49b, were synthesized with codon bias for expression in insect cells (Genscript). A further truncated form of Ino80 comprising residues 487–1556 was cloned with an N-terminal 8-histidine and C-terminal twin Strep-tag® II. Genes were cloned into transposition-compatible vectors using the MultiBac system (23). Genes were omitted as required for the production of mutant deletion complexes.

Complexes were expressed in BTI-TN-5B1-4 insect cells at 27°C for 60 h post-infection in Insect-XPRESS™ Protein-free Insect Cell Medium with L-glutamine (Lonza). Cells were harvested by centrifugation at 1000 × g for 15 min at 4°C. Purification used a three-step purification protocol. Following lysis by sonication in Buffer A [50 mM Tris, 1 mM TCEP, 2 mM benzamidine-HCl, 10% glycerol, pH 8.0] + 300 mM NaCl supplemented with 10 μl Benzonase® nuclease (Sigma-Aldrich) and one cOmplete Protease Inhibitor Tablet (Roche) per litre initial cell culture volume, lysates were clarified by centrifugation at 40 000 × g for 1 h at 4°C and filtered through a 0.45 μm filter before being loaded onto a cOmplete His-Tag Purification Column (Roche). The column was washed in Buffer A + 250 mM NaCl and eluted directly onto a StrepTactin HP column (GE Healthcare) with Buffer A + 250 mM NaCl and 200 mM imidazole. The StrepTactin-bound protein was then eluted with Buffer A + 200 mM NaCl and 5 mM desthiobiotin and applied to a HiTrap Q HP column and eluted with a gradient of 150–600 mM NaCl. The protein was concentrated to 2–3 μM in storage buffer containing 50 mM Tris, 250 mM NaCl, 1 mM TCEP and 10% glycerol (pH 8.0) and flash frozen in liquid nitrogen.

SC2 complex (N-8-His-Ino80_487-1556-twin Strep-tag® II-C plus Tip49a and Tip49b subunits) was purified using the same methods as all other complexes with an additional 2 mM MgCl_2_ in all purification buffers. This complex was additionally purified by size exclusion chromatography using a Superose 6 size exclusion column (GE Healthcare) in Buffer A.

‘Arp5 bypass’ (Arp5 [BP]) was generated by mutating the stop codon (TAA) to AAA, to allow read-through to a downstream stop codon. This generated a 43 residue extension at the C-terminus of Arp5 with the sequence KCGTGRWGRLTETRKETIPEGTRAMTAIKRQNKTHGCWVVSCS*, adding an additional 4.8 kDa to the mass of wild-type Arp5.

For production of a recombinant Arp5:Ies6 complex, codon optimized human Arp5 was re-cloned to append the coding sequence for a C-terminal double Strep-tag preceded by a Gly-Ser linker, to generate Arp5-2xSII. Arp5-2xSII and Ies6 were co-expressed in insect cells as described above. Cells were harvested by centrifugation at 1000 × g for 15 min at 4°C. Cells were lysed by sonication in Buffer A + 150 mM NaCl supplemented with 10 μl Benzonase® nuclease (Sigma-Aldrich) and 1 cOmplete Protease Inhibitor Tablet (Roche) per litre cell culture volume. The lysate was subsequently clarified by centrifugation at 40 000 × g for 1 h at 4°C and then filtered through a 0.45 μm filter before loading onto a StrepTactin HP column (GE Healthcare). Following extensive washing in Buffer A, the bound material was eluted in Buffer A + 150 mM NaCl and 5 mM desthiobiotin. Peak fractions were pooled and diluted slightly to a final NaCl concentration of <150 mM NaCl, then applied to a HiTrap Q HP column and eluted over a gradient of 50–1000 mM NaCl. Central peak fractions from the Q gradient were then pooled and concentrated to ∼500 μl for loading onto a Superdex 200 10/300 GL size exclusion column (GE Healthcare), pre-equilibrated in 50 mM Tris pH 8.0, 300 mM NaCl, 1 mM TCEP and 10% Glycerol. Concentrated aliquots (between 20–30 μM) of purified Arp5-2xSII:Ies6 were flash frozen in liquid nitrogen and stored at −80°C.

### Preparation of nucleosomes

All experiments utilized human nucleosomes reconstituted from histones expressed in *E. coli* and assembled on DNA fragments based on the Widom 601 positioning sequence (24). Human H2A and H2B or H2AZ and H2B were co-expressed in *E. coli*. Cells were lysed by sonication in buffer A (20 mM Tris pH7.5, 400 mM NaCl, 0.1 mM EDTA, 1 mM TCEP) plus Roche Protease Inhibitors (2 tablets per 100 ml). H2A/H2B or H2A/H2AZ dimers were purified as soluble proteins by HiTrap Q FF, HiTrap Heparin HP in Buffer A and eluted by a salt gradient by mixing with buffer B (20 mM Tris pH7.5, 2 M NaCl, 0.1 mM EDTA, 1 mM TCEP), followed by gel filtration on Superdex S200 in buffer B. Human H3.1 and H4 histones were co-expressed in *E. coli*, lysed in buffer A and purified as soluble tetramers on HiTrap Heparin HP in buffer A and eluted with a salt gradient, followed by Superdex S200 in buffer B. Histone octamers were prepared by mixing tetramers with a 10% molar excess of dimers and then purified by gel filtration on Superdex S200 in buffer B. Nucleosomes were reconstituted from octamers and DNA by salt gradient dialysis in several steps from 2 M to 0.2 M NaCl. The DNA fragment used was a 153 bp DNA fragment based on a 167 bp Widom fragment provided by Daniela Rhodes (25) that was further digested with HinfI. This core nucleosome was then modified in a variety of ways to produce different mononucleosome substrates.

For end-positioned nucleosomes with a 61 bp overhang, synthetic DNA (prepared from Oligo1 and Oligo2) was ligated onto the end and purified on a 5–20% glycerol gradient. After preparation, the purified nucleosomes were concentrated and buffer exchanged into 20 mM Tris pH7.5, 100 mM NaCl, 1 mM TCEP.

For end-positioned nucleosomes with a 101 bp overhang we used DNA fragment prepared from a plasmid containing 24 copies of the following sequence:

ATCGGCTGTGTGCACGAACCCCCCGTTCAGCC CGACCGCTGCGCCTTATCCGGTAACTATCGTCTT GAGTCCAACCCGGTAAGACACGACTTATCGCCAC CCCGAG TACATCGAT

Digestion with EcoRV releases a 115 bp fragment which was purified on HiPrep 16/60 Sephacryl S-400 HR column (GE Healthcare) in 20 mM Tris pH7.5, 250 mM NaCl. The fragment was dephosphorylated with Fast AP Thermosensitive Alkaline Phosphatase (ThermoFisher EF0652) and digested with AvaI (site underlined above). The 101 bp fragment was purified by ion-exchange chromatography on a HiTrap Q FF column (GE Healthcare) by a salt gradient (0.25–1 M NaCl) in 20 mM Tris pH7.5, 250 mM NaCl, 1 mM EDTA. The NaCl concentration was reduced to ∼50 mM during concentration of DNA in a Vivaspin concentrator with a 3 kDa cutoff. This fragment was then ligated onto the 153 bp nucleosome core particles described above.

End-positioned nucleosomes with an Alexafluor AF647 label on the short DNA tail were prepared by digesting the 153 bp DNA fragment with AccI and then replacing the small fragment by ligation with a synthetic DNA fragment prepared from Oligo3 and Oligo4 (below) to recreate a 153 bp fragment. This labeled DNA fragment was then used to reconstitute core nucleosomes by salt dialysis as described above.

Nucleosomes labeled on H3 were prepared from core nucleosomes containing a mutated H3.1 protein (C96V/C110A, R2C) which was labelled with Alexafluor AF555 C2-maleimide.

**Oligo 1**

5′ Phos –TCGGGGTGGCGATAAGTCGTGTCTTACCGGGTTGGACTCAAGACGATAG

TTACCGGATAAGGCGC

**Oligo2**

5′-GCGCCTTATCCGGTAACTATCGTCTTGAGTCCAACCCGGTAAGACACGACTTA

TCGCCACC

**Oligo3**

5′ Alexa647 -AATCCCGGTGCCGAGGCCGCTCAATTGGTCGT

**Oligo4**

5′ Phos -CTACGACCAATTGAGCGGCCTCGGCACCGGGATT

### ATPase assays

ATPase activity of the complex was measured by a coupled assay that measures ADP release as described previously (26) but utilizing NADH fluorescence rather than absorbance to increase sensitivity. A final concentration of 100 μM NADH, 0.5 mM phosphoenolpyruvate, 100 U/ml pyruvate kinase (Sigma), 20 U/ml lactate dehydrogenase (Sigma) were used in all reactions in a final volume of 50 μl. Reactions were conducted using 10 nM purified Ino80 complex, 100 nM hH2A-containing 250 bp end-positioned nucleosome and 1 mM ATP unless otherwise stated. ATP solutions were made with a molar ratio of 1:1:2 ATP:MgCl_2_:TRIS base. IP_6_ was found to decrease the free concentration of Mg^2+^, so all experiments using IP_6_ were conducted in the presence of a vast excess of Mg^2+^ (5 mM Mg^2+^ with up to 250 μM IP_6_). It was also noted that a precipitate formed when concentrated Mg^2+^ was added to concentrated IP_6_, which could be prevented through paying attention to the order of addition. Single-point IP_6_-inhibited reactions were carried out with 250 μM IP_6_. Reactions were conducted by mixing all components immediately prior to transferring to an Optiplate-384 Black Opaque 384-well microplate (Perkin Elmer) that had been pre-incubated at 37°C. Reactions were monitored fluorescently using an excitation of 335 nm and an emission of 469 nm at 37°C with a Clariostar microplate reader (BMG Labtech). All reaction rates were determined using the maximum initial linear rate and reaction kinetics were analyzed assuming a Michaelis–Menten model.

### Nucleosome sliding assays

A gel-based assay was used essentially as described previously (17,18). End-positioned nucleosomes with a 61 bp overhang were prepared as described above. For a typical assay, frozen stocks of hIno80 were rapidly thawed and diluted to 10x working concentration (1 μM) in Assay Buffer (25 mM HEPES, 50 mM NaCl and 1 mM TCEP, pH 8.0) and then pre-incubated at 37°C with human H2A- or H2A.Z-containing nucleosomes at a final concentration of 300 nM. For time course assays, a reaction mix of appropriate volume was prepared to cover each time point. Reactions were started with the addition of ATP and MgCl_2_ to a final concentration of 1 and 2 mM, respectively and run for up to 10 min at 37°C and. Reactions were terminated by addition of EDTA (5 mM) and unlabeled nucleosomes (1 μM). Reaction products were resolved by native gel electrophoresis using 6% acrylamide-TBE gels, run at 80 V for 120 min at 4°C. Gels were visualized and digitized for quantification using a BioRad Chemidoc MP system.

FRET-based microtiter assays for measuring nucleosome sliding by recombinant Ino80 complexes were performed on a CLARIOstar® microplate reader (BMG Labtech) in a final volume of 50 μl in OptiPlate-384 Black Opaque 384-well microplates (Perkin Elmer). Human H2A-containing, end-positioned nucleosomes (with a 61 bp overhang and labeled on histone H3 with AlexaFluor 555 (AF555) (Life Technologies) and AlexaFluor 647 (AF647) (Life Technologies) on the 5’ DNA end closest to the histone core) were prepared in FRET-A buffer (25 mM HEPES, 50 mM NaCl and 1 mM TCEP, pH 8.0). For a standard assay, the final working concentrations of nucleosome and Ino80 core complex were 300 and 100 nM, respectively. Reactions were initiated with the injection of ATP to a final concentation of 1 mM using the built-in reagent injectors, which were pre-filled with 10x ATP solution (FRET-A buffer plus 10 mM ATP, 20 mM MgCl_2_). The sliding reaction was monitored via the decrease in fluorescence of AF647 (excitation of AF555 at 535 nm and emission of AF647 at 680 nm), with readings taken every 20 s.

### Nucleosome binding assays

Microscale thermophoresis (MST) (27) was used to determine binding constants for nucleosome binding to Ino80 complexes. Ino80 core complex-nucleosome interactions were measured on a temperature equilibrated Monolith™ NT.115 (NanoTemper), using a fluorescent human H2A-containing nucleosome labeled on histone H3 with AlexaFluor 647 (Life Technologies). For each set of measurements, a two fold serial-dilution of wild-type Ino80 core complex or a mutant variant was prepared at 2x final concentration (starting at 2 μM) in MST-Buffer [25 mM HEPES, 50 mM NaCl, 1 mM TCEP, 10% Glycerol, pH 8.0] before mixing with an equal volume of nucleosome stock solution (also in MST-Buffer), to yield a final nucleosome concentration of 100 nM. ATP, ATPγS or ADP-NP (Sigma-Aldrich) were added to a final concentration of 1 mM as required by the experiment. IP_6_ (Sigma-Aldrich) was added to a final concentration of 2 mM. Samples were given 15 min to equilibrate before being loaded into Monolith™ NT.115 MST Premium Coated capillaries (NanoTemper). The system was given a further 15 min to re-equilibrate at 37°C before beginning each scan. LED Power was set at 30% for all experiments, while MST power was set to 15% for the set of experiments with the different complexes and 80% for the set of experiments with different ligands. All other settings were left at the default values. K_d_ fitting was performed on the NT Analysis software (NanoTemper) using the temperature jump data only.

### SEC-MALS

Protein samples for multi-angle light scattering (MALS) were further purified by size exclusion chromatography (SEC) with a Superose 6 10/30 column (GE). Fractions corresponding to the first half of the elution peak were pooled and concentrated. SEC-MALS was performed using a Superose 6 10/30 column (GE) at room temperature running at 0.5 ml/min. Light scattering analysis was performed with a miniDAWN TREOS light scattering detector (WYATT) and the refractive index was measured using an Optilab T-rEX refractometer.

## RESULTS

### Expression of recombinant hIno80 in insect cells

**Figure 1.**
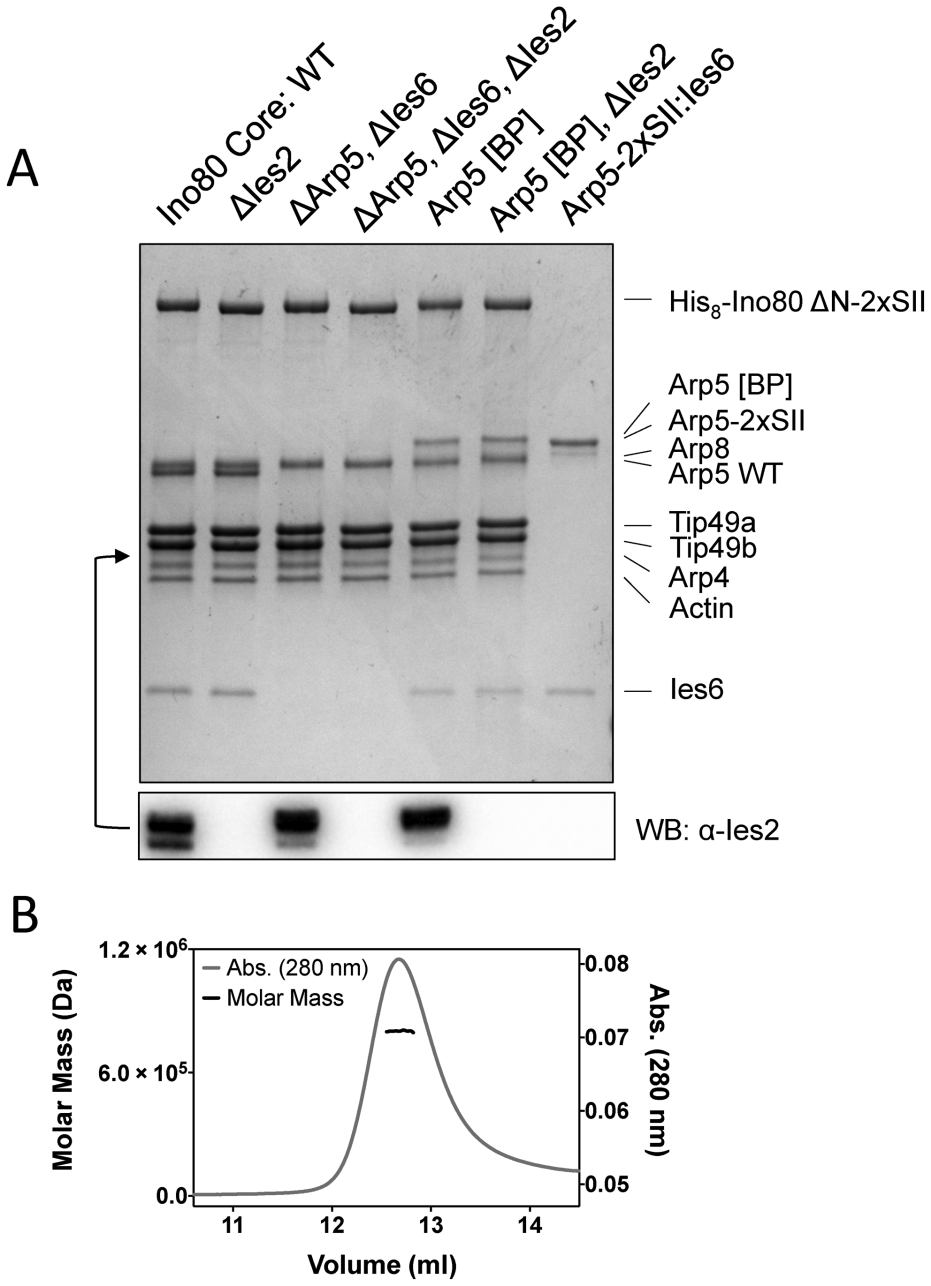
(**A**) Coomassie-stained SDS gel of protein complexes used in this study. Subunit identities are as marked. Western blot analysis for Ies2 is shown below and the band is situated as indicated by the arrow. (**B**) SEC-MALS analysis of hIno80 core complex. The expected mass of a complex containing one subunit each of actin, Arp4, Arp5, Arp8, Ies2, Ies6 and affinity tagged Ino80 together with three copies each of Tip49a and Tip49b is 743 583 Da. The molecular weight determined by SEC-MALS is 802 000 ± 10 400 Da.

Previous work on yeast and human Ino80 complexes has used endogenous protein extracted from cells using a tagged Ino80 subunit (17,18). However, expression of Ino80 complex is low in both yeast and human cells so we decided to circumvent this by expressing recombinant human complex in insect cells. To simplify the system we reduced the complex to a conserved ‘core’ of subunits that are present across different species (1). We also cloned a truncated Ino80 subunit that lacks the N-terminal region of the complex that has been shown to have a regulatory role and to be dispensable for activity (19). We used the MultiBac expression system (23) to produce a core complex in insect cells that comprised a truncated Ino80 subunit (residues 267–1556 with an N-terminal 8-histidine and C-terminal twin Strep-tag® II) together with actin, Arp4, Arp5, Arp8, Ies2, Ies6, Tip49a and Tip49b (Figure 1A). Identities of the bands on the SDS gel were confirmed by mass spectrometry. The Ies2 subunit was difficult to detect on SDS gels because it lies in a region close to the Tip49b and Arp4 bands, so the presence of the protein was confirmed by Western blot (Figure 1A). This system consistently yields over 0.5 mg of purified complex from two liters of insect cells. We also produced a subcomplex of hIno80 (SC2) comprising residues 487–1556 of the main subunit in complex with the Tip49a and Tip49b subunits (Supplementary Figure S1).

Studies of the stoichiometry of the subunits in the complex have been hampered by a lack of sufficient material for most biophysical techniques. Our recombinant protein has allowed us to prepare sufficient amounts to undertake analysis by size exclusion chromatography and multi-angle light scattering (SEC-MALS) (Figure 1B). These data gave a molecular weight estimate for the complex of 802 ± 1% kDa. The calculated molecular weight for a complex containing three copies each of the Tip49 proteins together with one copy of each of the other proteins is 744 kDa, in good agreement with our measured value. The errors on the weight estimate we obtain are sufficiently low to preclude the presence of two Tip49 hexamers per complex.

### Nucleosome sliding assays

**Figure 2.**
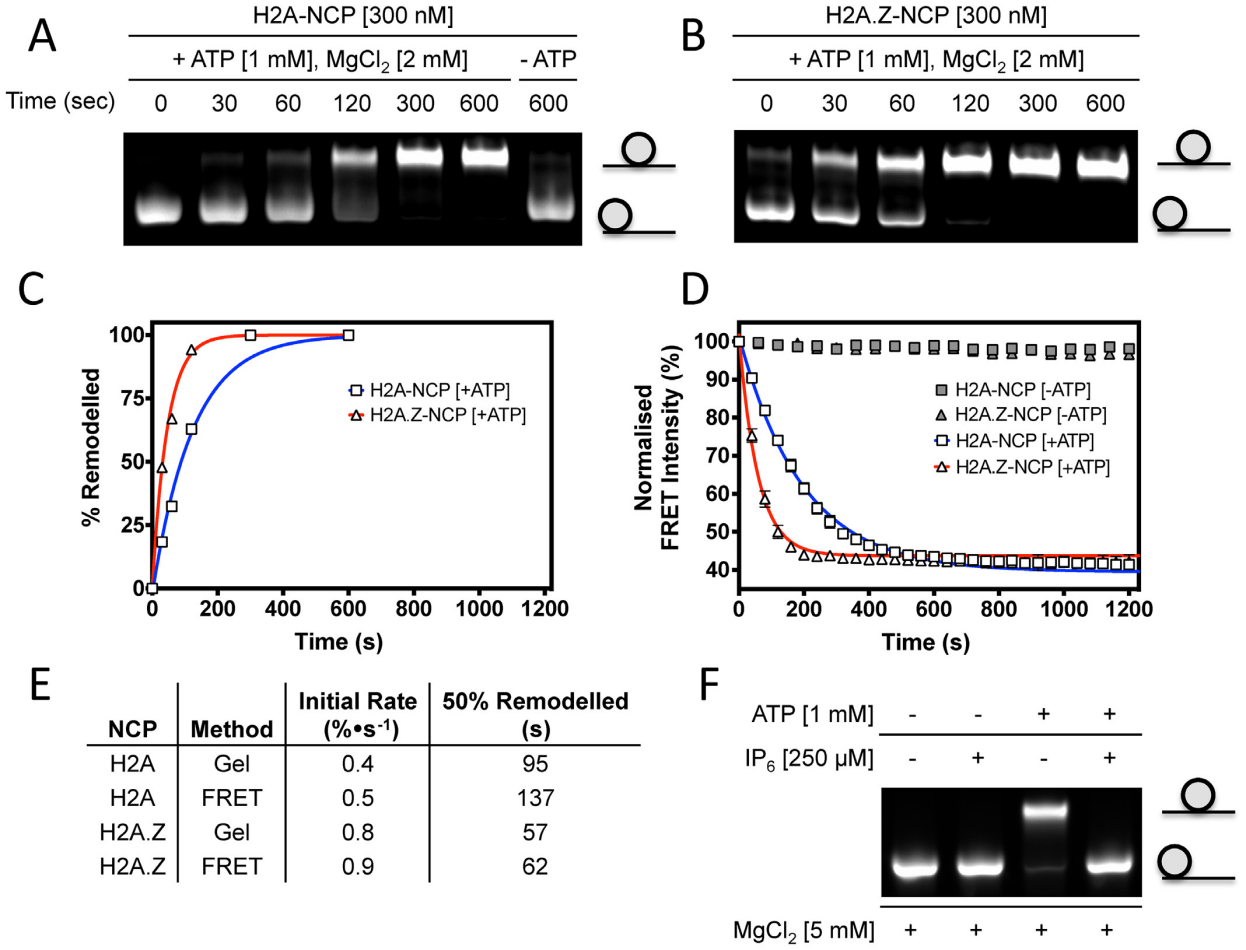
Mononucleosome sliding assays. (**A**) Gel-based assay with nucleosomes containing H2A. (**B**) as (A) but with nucleosomes containing H2AZ. (**C**) Quantification of the gels in (A) and (B). (**D**) FRET-based sliding assays using the same nucleosome substrates. (**E**) Rates of nucleosome sliding determined by each assay. (**F**) Inhibition of nucleosome sliding by IP_6_.

Both yeast and human Ino80 complexes have been shown to have nucleosome sliding activity (17,18). Using a standard gel-based assay, that separates end positioned and centrally positioned nucleosomes on the basis of their differing electrophoretic mobility (28–30), our recombinant hIno80 also catalyses nucleosome sliding of end-positioned mononucleosomes towards the middle of DNA fragments (Figure 2A and B). It has been shown previously (21) that inositol hexaphosphate (IP_6_) inhibits the ATPase activity of the yeast Ino80 complex and hence also inhibits the sliding activity. Nucleosome sliding by our recombinant Ino80 is also inhibited by IP_6_ (Figure 2F). The hIno80 shows a slightly higher rate of sliding for nucleosomes containing H2AZ histones compared to those containing canonical H2A histone (Figure 2).

Although the gel-based nucleosome sliding assay provides a simple method to measure activity, we developed a continuous time point assay using FRET based system in a microtiter plate reader (Figure 2C and D). The results for nucleosome sliding obtained with this assay are similar to those from the gel-based assay (Figure 2A–E) but with continuous time point measurements so provides a more quantifiable method to assay initial rates of nucleosome sliding. By using DNA overhangs of different lengths, we were able to demonstrate experimentally that the maximum FRET distance we can monitor is available with an overhang of 60 bp (data not shown). Sliding of nucleosomes with longer overhangs can still be monitored with this assay but only the initial rates can be measured as the signal is lost once the nucleosomes have been shifted by 30–40 bp. We therefore use a nucleosome with 60 bp overhang for our FRET assays to remain within this limit.

### Nucleosome binding

**Figure 3.**
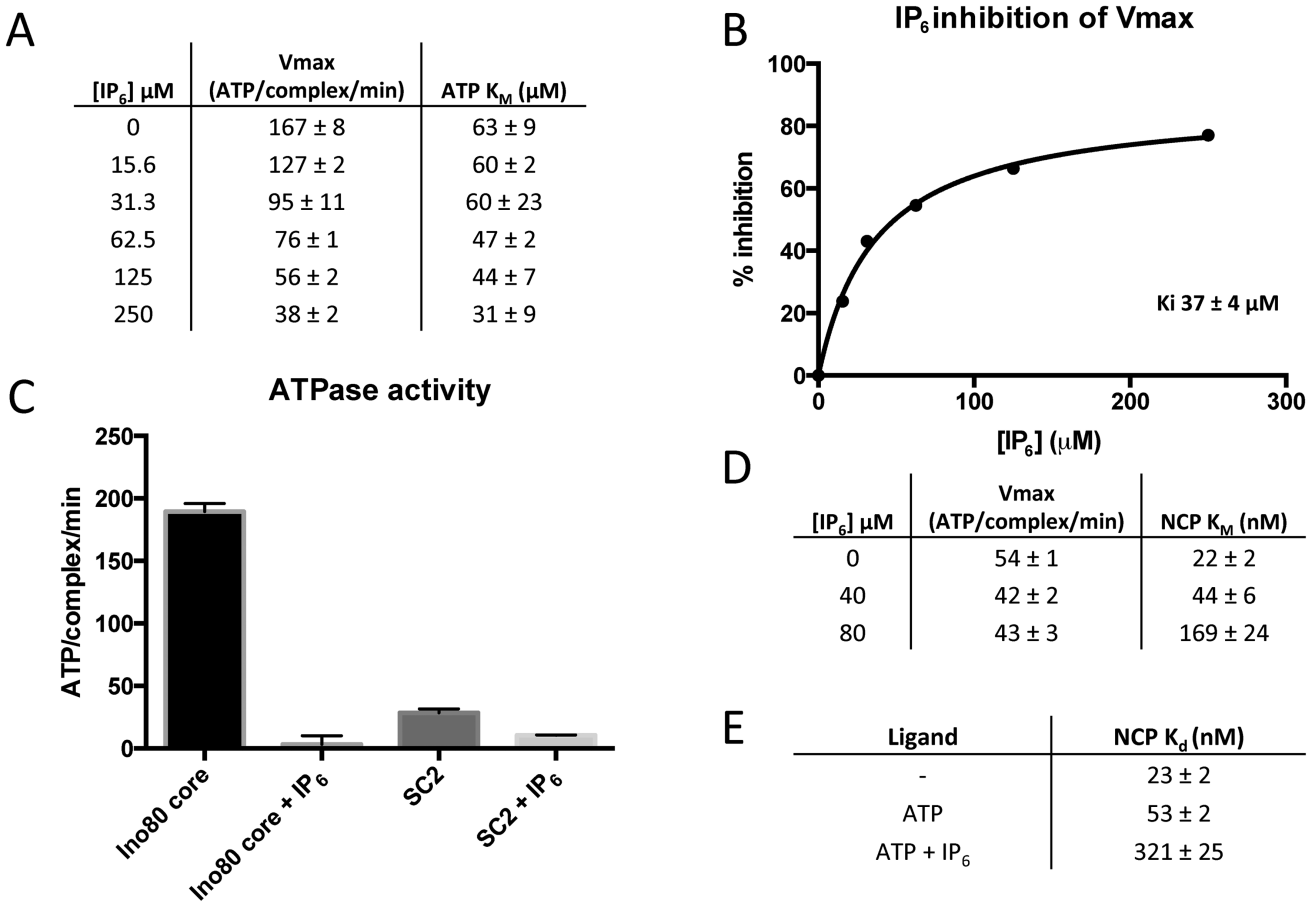
IP_6_ inhibition mechanism. (**A**) ATPase activity of Ino80 over a range of IP_6_ and ATP concentrations. Nucleosome concentration was kept constant at 100 nM for all of these assays. (**B**) V_max_ values from (A) plotted as % inhibition versus IP_6_ concentration, with 0% inhibition equal to where [IP_6_] is 0 μM. (**C**) Single point inhibition of Ino80 and SC2. Assays were conducted using 250 μM IP_6_, 1 mM ATP and 100 nM nucleosome. (**D**) ATPase activity of Ino80 over a range of nucleosome and IP_6_ concentrations. The ATP concentration was 1 mM for these assays. (**E**) MST binding data of Ino80 binding nucleosome in the presence and absence of different ligands. The ATP concentration was 1 mM and the IP_6_ concentration was 250 μM. These binding experiments were conducted in the presence of 5 mM MgCl_2_.

Binding of nucleosomes to the hIno80 was determined by MST (Table 1 and Supplementary Figure S2). The core complex associates with human nucleosomes with a Kd of 20 nM (Figure 3), comparable to that reported previously by other groups for endogenous yeast Ino80 complex using gel-shift data (20). Our hIno80 showed slightly lower affinity for nucleosomes containing H2AZ than H2A (Table 1) despite being able to slide them slightly more efficiently (Figure 2).

**Table 1.** Binding affinities for mononucleosome binding with different complexes as determined by MST

Complex	NCP	K_d_ (nM)
Wild Type	H2A	20.7 ± 1.0
Wild Type	H2A.Z	54.1 ± 4.7
ΔArp5, ΔIes6	H2A	106.0 ± 6.1
ΔIes2	H2A	29.7 ± 2.0

### ATPase activity

The ATPase rates obtained were similar to values reported previously for cognate yeast and human Ino80 complexes (20,31,32) (Table 2 and Supplementary Figure S3). The K_M_ for nucleosomes was comparable to the K_d_ determined by MST (Figure 3). In the presence or absence of saturating concentrations of nucleosomes, the K_M_ for ATP was virtually unchanged (Table 2 and Supplementary Figure S4) but the ATPase rate was stimulated over 50-fold.

**Table 2.** ATPase data

A			
Complex	Basal ATPase Rate (ATP/complex/min)	Basal ATP K_M_ (μM)	
WT	1.7 ± 0.1	323 ± 55	
ΔIes2	2.8 ± 0.1	273 ± 36	
ΔArp5, ΔIes6	2.4 ± 0.2	225 ± 63	
ΔArp5, ΔIes6, ΔIes2	1.2 ± 0.2	244 ± 116	
Arp5 [BP]	1.0 ± 0.1	171 ± 55	
Arp5 [BP], ΔIes2	7.5 ± 0.4	326 ± 44	
Basal (non-nucleosome stimulated) ATPase kinetic data for the various Ino80 complexes.			

B			
Complex	Stimulated ATPase Rate (ATP/complex/min)	Stimulated NCP K_M_ (nM)	ATPase Stimulation Factor
WT	97 ± 3	9 ± 2	57
ΔIes2	9.7 ± 0.7	47 ± 11	3
ΔArp5, ΔIes6	83 ± 3	39 ± 5	35
ΔArp5, ΔIes6, ΔIes2	4.9 ± 0.3	25 ± 7	4
Arp5 [BP]	200 ± 5	8 ± 1	200
Arp5 [BP], ΔIes2	53 ± 2	12 ± 2	7

Nucleosome stimulated ATPase kinetic data for the various Ino80 complexes. These experiments were performed in the presence of 1 mM ATP over a range of nucleosome concentrations. Stimulation factor refers to the ratio of the stimulated to basal ATPase rates.

### IP_6_ inhibits hIno80 nucleosome sliding and ATPase stimulation by reducing nucleosome affinity

It has been shown that inhibition of nucleosome sliding by endogenous yeast Ino80 complex by IP_6_ is a consequence of inhibition of the ATPase activity of the complex (21). IP_6_ shows the same effect on our recombinant hIno80, inhibiting both nucleosome sliding (Figure 2F) and ATPase activity (Figure 3A–C) with a K_i_ for IP_6_ of 37 ± 4 μM (Figure 3B and Supplementary Figure S5). We investigated the basis of this inhibition of ATPase activity by IP_6_ to understand a little more about the mechanism. Our data show that when titrating ATP in the presence of nucleosomes, the V_max_ of hIno80 decreases as the IP_6_ concentration increases while the K_M_ remains relatively unchanged (in fact, if anything becomes a little tighter (Figure 3A)) demonstrating non-competitive inhibition with respect to ATP. Conversely, when titrating nucleosomes in the presence of saturating ATP, the V_max_ remains relatively unchanged over an increasing concentration range of IP_6_, while the K_M_ increases (Figure 3D and Supplementary Figure S6) demonstrating competitive inhibition with respect to nucleosomes. Prompted by these observations, we then performed nucleosome binding experiments in the presence of IP_6_ and ATP. We observed a more than 10-fold decrease in the affinity for NCP in the presence of IP_6_ and ATP (Figure 3E and Supplementary Figure S7). Hence, IP_6_ is a non-competitive inhibitor of hIno80 ATPase and binds at an allosteric site rather than the ATP-binding site. Consequently, the ‘inhibition’ of ATPase activity is actually due to blocking stimulation by reducing nucleosome affinity.

Finally, we were able to show that even a minimal sub-complex of hIno80 containing just the Tip49 subunits and the C-terminal portion of the Ino80 subunit (SC2) (residues 487–1556) is still inhibited by IP_6_ (Figure 3C), ruling out the remainder of the complex as containing the binding site for IP_6_. Furthermore, since the Tip49 proteins are present in other complexes such as yeast Swr1 that are not inhibited by IP_6_, this suggests strongly that the IP_6_ binding site is located in the C-terminal region of Ino80, or at the interface with the Tip49 proteins, and acts in an allosteric manner to inhibit ATPase activity in hIno80.

### Ies2 subunit regulates ATPase activity

Having established a set of assays to monitor the basic activities of the core complex, we then prepared a series of complexes that lacked specific subunits (Figure 1A). Previous work on human and yeast Ino80 complexes (15,32,33) have shown the importance of the Arp5/Ies6 and Ies2 subunits for sliding activity and that Ies2 has a role in regulating ATPase activity (32). However, the molecular mechanism for this process was not determined. For yeast Ino80 complex, deletion of Ies2 results in a loss of the ability to recruit the Arp5 and Ies6 subunits (33). By contrast, for our recombinant human core complex, Ies2 can be deleted without disrupting assembly of the Arp5/Ies6 subunits (Figure 1A). Endogenous Ino80 complex prepared from human cells that are depleted in Ies2 can also retain Arp5/Ies6 within the complex (32).

**Figure 4.**
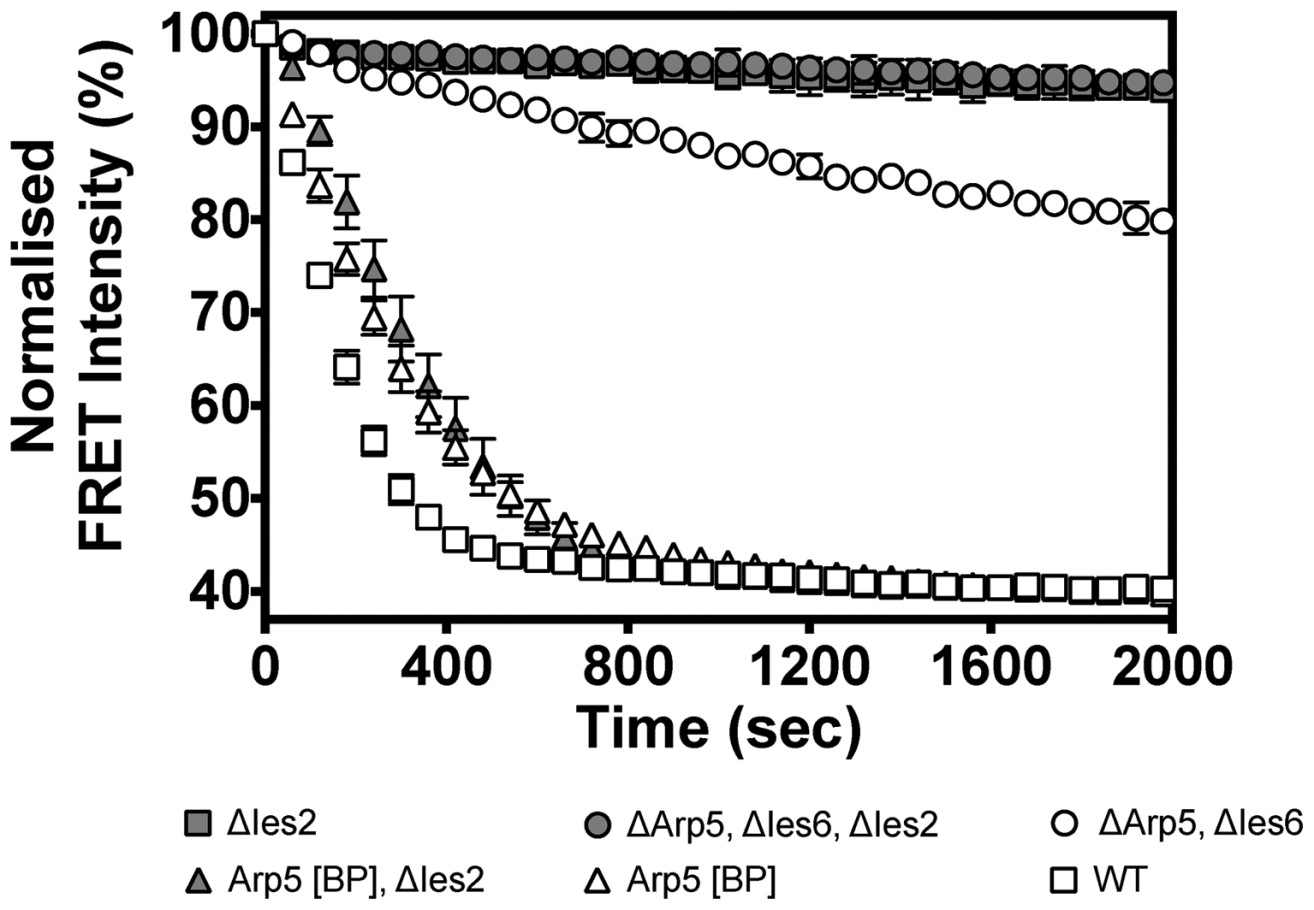
FRET-based assay of nucleosome sliding activity of mutant complexes.

The hIno80ΔIes2 complex was deficient in nucleosome sliding (Figure 4) but had a similar K_M_ for nucleosomes as the wild-type complex (Table 2). The lack of sliding activity was correlated with a commensurate loss of ATPase activity that was due to a significantly reduced stimulation of ATPase activity in response to bound nucleosome, compared to the core complex (Table 2). In fact, in every complex we prepared that lacks Ies2, the stimulation of ATPase activity by nucleosomes was minimal (Table 2) confirming the role of this subunit in regulating ATPase activity in the complex (15,32,33).

### Arp5/Ies6 subunits couple ATP hydrolysis to nucleosome sliding

**Figure 5.**
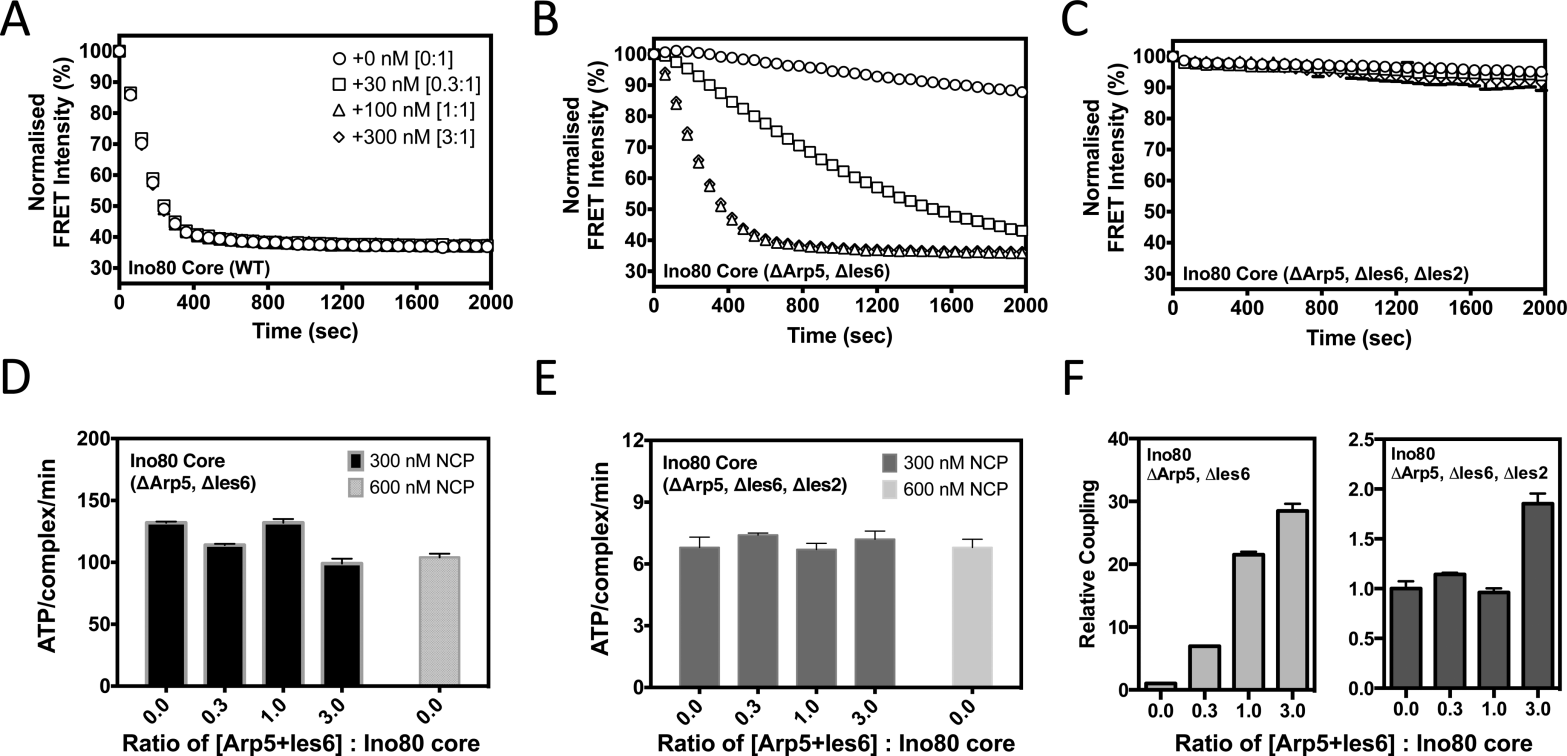
Arp5/Ies6 couple ATPase to sliding activity. FRET-based sliding assay of complexes after supplementation with increasing levels (0, 30, 100, 300 nM) of tagged-Arp5/Ies6, at a fixed Ino80 complex concentration (100 nM). (**A**) Wild-type Ino80 core complex (symbols are the same in panels A–C). (**B**) Ino80ΔArp5Ies6, (**C**) Ino80ΔIes2Arp5Ies6, (**D**) ATPase rates for Ino80ΔArp5Ies6 complex in the same ratios as (B) with 300 nM nucleosomes (dark grey) or 600 nM nucleosomes (light grey), (**E**) similar data for hIno80ΔIes2Arp5Ies6, (**F**) coupling ratios for the complexes using the data in panels (B–E). Relative coupling = (Sliding rate/min)/(ATP/complex/min). These values were then scaled for the assay with the ratio of 0.0 Arp5+Ies6 : Ino80 core to have a relative coupling value of 1 within each complex data set.

Deletion of either Arp5 or Ies6 produced a complex that lacked both subunits indicating a close interaction between these proteins as shown previously (15,32,33), so we deleted these as a pair in the expression constructs. Although the mutant complex bound nucleosomes about 5-fold less tightly, once saturated, the stimulated ATPase rate was almost the same as the core complex (Table 2). However, the nucleosome sliding activity of the complex was severely impaired (Figures 4 and 5A–C). The degree of coupling (ratio of nucleosome sliding : ATPase activity) was less than 10% of that observed for the core complex (Figure 5D and F) suggesting a role for Arp5/Ies6 in coupling nucleosome-stimulated ATPase activity to sliding.

### Reconstitution of complexes with tagged, purified Arp5/Ies6

Complexes lacking Arp5/Ies6 were tested for their ability to reconstitute activity in the presence of added purified Arp5/Ies6. Purified protein was added to the sample and assayed for ATPase and nucleosome sliding (Figure 5A, B and D). As expected, addition of Arp5/Ies6 to the wild-type complex had no effect on either activity (Figure 5A). However, quite a different result was obtained with the hIno80ΔArp5/Ies6 complex (Figure 5B and D). As Arp5/Ies6 was added, the ATPase rate remained unchanged but the nucleosome sliding increased. This effect was at a maximum when the Arp5/Ies6 was equimolar with the complex (Figure 5B and D). Hence, this is a direct demonstration of the role of Arp5/Ies6 in coupling of ATPase to sliding activity. When similar experiments were performed in which the Ies2 subunit was also deleted (hIno80ΔArp5/Ies6/Ies2), the coupling effects were lost (Figure 5C, E and F) due to the greatly reduced ability of this complex to respond to nucleosome binding (Table 2).

### Requirement for Ies2 can be overcome by an Arp5 ‘bypass’ mutation

While we were doing some controls to test the effect of placing a C-terminal tag on the Arp5 subunit, we substituted the tag with an additional 43 residues created instead by mutating the stop codon in the wild-type Arp5 gene to allow read through to an alternative stop (hereafter referred to as Arp5[BP]). The complex was then prepared with the wild-type Arp5 subunit replaced by Arp5[BP]. This complex had ATPase and sliding activities that were similar to wild-type complex (Table 2 and Figure 4). However, we also prepared complex containing this mutated Arp5 but lacking Ies2. To our surprise, this complex retained essentially the same activities as complex containing Ies2 (Table 2 and Figure 4). Consequently, the presence of the additional 43 residues at the end of the Arp5 protein allows the complex to bypass the requirement for the Ies2 subunit for stimulation of ATPase activity but also couples the elevated ATPase activity to sliding, further emphasising this role for Arp5 in the hIno80 complex.

## DISCUSSION

Endogenous Ino80 complex prepared from human cells contains at least 14 subunits (1,18). Of these, there are at least 9 conserved in yeast that contains a total of 15 subunits (1,17). Many of the roles played by conserved and species-specific subunits are unknown. We have successfully expressed and purified a core complex of human Ino80 in insect cells that is fully active in nucleosome sliding.

The recombinant core complex contains one heterohexamer of the Tip49 proteins together with a single copy of each of the other conserved subunits. Although some structural analyses have suggested that Tip49 heterohexamers in isolation might associate to form dodecameric double rings both in solution and in crystals (5–7,11), we see no evidence for that in our purified protein and conclude that the formation of dodecamers is likely an artefact of a high concentrations of the free protein in the absence of protein partners. Indeed, it has been reported that the presence of introduced affinity tags might affect association of hexamers (12) and dodecamers still form even when the domain that is proposed to be the hexamer interface is deleted (6). Although one report suggested dodecamers in the low resolution structure of Ino80 complex (16) other studies on both Ino80 complex and the related Swr1 complex (14,15) suggest a single heterohexamer. The data we present here are consistent with this latter proposal.

We have designed a FRET-based assay for nucleosome sliding that allows real-time monitoring of sliding up to 30 bp. This has provided us with a convenient method for quantifying this activity. The gel-based nucleosome mobility assays fulfil a different role because they provide information about intermediates between the end-positioned and centrally located nucleosomes whereas the FRET method only provides an estimate of the bulk average within the assay so therefore gives an estimate of the bulk progression of the nucleosomes from the end toward the centre of the DNA. The method also has applications for single-molecule experiments that are underway.

The ATPase activity of both yeast and human Ino80 complex is stimulated by nucleosomes (20,31,32). We show that the hIno80 is similarly stimulated (around 50-fold) and also that the K_M_ for ATP is essentially unaltered with or without nucleosomes present. Hence, stimulation is entirely a consequence of an increased rate of catalytic turnover of ATP. This is reminiscent of the stimulation of ATPase activity by ssDNA in Superfamily 1 helicases that was shown to be due to improved binding of the essential magnesium ion co-factor (34). Further experiments will be required to determine whether this is also the case for Ino80, which is a Superfamily 2 helicase/translocase family member.

We then carried out similar assays in the presence and absence of IP_6_. The ATPase activity of hIno80 is stimulated 50-fold by nucleosomes which is a direct stimulation of V_max_ but leaves the K_M_ for ATP essentially unaltered (Table 2). We discovered that IP_6_ acts by blocking the stimulation of V_max_ by preventing nucleosome binding, rather than competitively inhibiting ATPase. Furthermore, we show that a complex that comprises just the Rvb subunits and the C-terminal portion of Ino80 is inhibited by IP_6_. Since other complexes that contain the Rvb proteins are not inhibited by IP_6_, this suggests the IP_6_ binding site is located in the C-terminal region of the Ino80 subunit. An allosteric regulation mechanism would also be supported by the observation that the Swi/Snf complex is activated by inositol phosphates (21).

It has been reported previously that human Ino80 complex is regulated by an interplay between the Arp5/Ies6 and Ies2 subunits (32). The Ies2 subunit acts as a molecular throttle to couple binding of nucleosome to relief of an auto-inhibition of ATPase activity in the complex. By contrast, the Arp5/Ies6 pair act to couple ATP hydrolysis to nucleosome sliding. Consequently, in the absence of Arp5/Ies6, the complex responds normally to the binding of nucleosomes by enhancing the ATPase activity, but is uncoupled from sliding. We show that as Arp5/Ies6 is titrated back into a system that lacks these components, the ATPase essentially remains unaltered but the coupling of sliding to ATP hydrolysis is enhanced in a proportion relating to the stoichiometry of the Arp5/Ies6 subunits within the complex. This is direct evidence that the Arp5/Ies6 module provides the ability to couple ATP hydrolysis to nucleosome sliding.

A role for Arp5 in coupling ATPase and sliding activities was also highlighted by the Arp5[BP] mutant. Extension of the C-terminal region of Arp5 creates a gain of function in hIno80, such that the complex is able to bypass the requirement for regulation by the Ies2 subunit. This suggests an interaction between the C-terminal region of Arp5 and the Ino80 subunit that is disrupted in our mutant protein. The crystal structure of Arp7/Arp9 bound to HSA domain of Sth1 (35) reveals that the Arp7 and Arp9 proteins both interact with a long helical section of the Sth1 subunit via a hydrophobic groove close to the C-terminus of the proteins in a manner similar to the interaction between actin and a number of its ligands (36). The analogy with the Arp7, Arp9 and actin proteins suggests the hydrophobic groove in Arp5 as a likely binding site for some helical part of the Ino80 protein. However, this cannot be the whole story because the addition of non-specific protein mass at the C-terminus of Arp5 activates the ATPase activity of the complex but in a way that, unlike the stimulation by Ies2, is coupled to nucleosome sliding. This suggests a steric role for Ies2 that acts to block a conformational change that would inhibit the ATPase. Blocking of this change, either by Ies2 or the C-terminal extension of Arp5, thereby stimulates the ATPase and this activity is then coupled to nucleosome sliding by Arp5/Ies6.

## Supplementary Material

SUPPLEMENTARY DATA

## References

[1] Clapier C.R., Cairns B.R. (2009). The biology of chromatin remodelling complexes. Ann. Rev. Biochem..

[2] Singleton M.R., Dillingham M.S., Wigley D.B. (2007). Structure and mechanism of helicases and nucleic acid translocases. Annu. Rev. Biochem.

[3] Downs J.A., Allard S., Jobin-Robitaille O., Javaheri A., Auger A., Bouchard N., Kron S.J., Jackson S., Côté J. (2004). Binding of chromatin-modifying activities to phosphorylated histone H2A at DNA damage sites. Mol. Cell.

[4] Saravanan M., Wuerges J., Bose D., McCormack E.A., Cook N.J., Zhang X., Wigley D.B. (2012). Interactions between the nucleosome histone core and Arp8 in the INO80 chromatin remodeling complex. Proc. Natl. Acad. Sci. U.S.A..

[5] Matias P.M., Gorynia S., Donner P., Carrondo M.A. (2006). Crystal structure of the human AAA+ protein RuvBL1. J. Biol. Chem..

[6] Gorynia S., Bandeiras T.M., Pinho F.G., McVey C.E., Vonrhein C., Round A., Svergun D.I., Donner P., Matias P.M., Carrondo M.A. (2011). Structural and functional insights into a dodecameric molecular machine - the RuvBL1/RuvBL2 complex. J. Struct. Biol..

[7] Lakomek K., Stoehr G., Tosi A., Schmailzl M., Hopfner KP. (2015). Structural basis for dodecameric assembly states and conformational plasticity of the full-length AAA+ ATPases Rvb1 · Rvb2. Structure.

[8] Vorobiev S., Strokopytov B., Drubin D.G., Frieden C., Ono S., Condeelis J., Rubenstein P.A., Almo S.C. (2003). The structure of nonvertebrate actin: implications for the ATP hydrolytic mechanism. Proc. Natl. Acad. Sci. U.S.A..

[9] Fenn S., Breitsprecher D., Gerhold C.B., Witte G., Faix J., Hopfner K.P. (2011). Structural biochemistry of nuclear actin-related proteins 4 and 8 reveals their interaction with actin. EMBO J..

[10] Gerhold C.B., Winkler D.D., Lakomek K., Seifert F.U., Fenn S., Kessler B., Witte G., Luger K., Hopfner K.P. (2012). Structure of Actin-related protein 8 and its contribution to nucleosome binding. Nucleic Acids Res..

[11] Torreira E., Jha S., López-Blanco J.R., Arias-Palomo E., Chacón P., Cañas C., Ayora S., Dutta A., Llorca O. (2008). Architecture of the pontin/reptin complex, essential in the assembly of several macromolecular complexes. Structure.

[12] Cheung K.L., Huen J., Kakihara Y., Houry W.A., Ortega J. (2010). Alternative oligomeric states of the yeast Rvb1/Rvb2 complex induced by histidine tags. J. Mol. Biol..

[13] Ewens C.A., Su M., Zhao L., Nano N., Houry W.A., Southworth D.R. (2016). Architecture and nucleotide-dependent conformational changes of the Rvb1-Rvb2 AAA+ complex revealed by cryoelectron microscopy. Structure.

[14] Nguyen V.Q., Ranjan A., Stengel F., Wei D., Aebersold R., Wu C., Leschziner A.E. (2013). Molecular architecture of the ATP-dependent chromatin-remodeling complex SWR1. Cell.

[15] Watanabe S., Tan D., Lakshminarasimhan M., Washburn M.P., Hong E-J.E., Walz T., Peterson C.L. (2015). Structural analyses of the chromatin remodelling enzymes INO80-C and SWR-C. Nat. Commun..

[16] Tosi A., Haas C., Herzog F., Gilmozzi A., Berninghausen O., Ungewickell C., Gerhold C.B., Lakomek K., Aebersold R., Beckmann R. (2013). Structure and subunit topology of the INO80 chromatin remodeler and its nucleosome complex. Cell.

[17] Shen X., Mizuguchi G., Hamiche A., Wu C. (2000). A chromatin remodelling complex involved in transcription and DNA processing. Nature.

[18] Jin J., Cai Y., Yao T., Gottschalk A.J., Florens L., Swanson S.K., Gutiérrez J.L., Coleman M.K., Workman J.L., Mushegian A. (2005). A mammalian chromatin remodeling complex with similarities to the yeast INO80 complex. J. Biol. Chem..

[19] Chen L., Cai Y., Jin J., Florens L., Swanson S.K., Washburn M.P., Conaway J.W., Conaway R.C. (2011). Subunit organization of the human INO80 chromatin remodeling complex: an evolutionarily conserved core complex catalyzes ATP-dependent nucleosome remodeling. J. Biol. Chem..

[20] Udugama M., Sabri A., Bartholomew B. (2011). The INO80 ATP-dependent chromatin remodeling complex is a nucleosome spacing factor. Mol. Cell. Biol..

[21] Shen X., Xiao H., Ranallo R., Wu W.H., Wu C. (2003). Modulation of ATP-dependent chromatin-remodeling complexes by inositol polyphosphates. Science.

[22] Steger D.J., Haswell E.S., Miller A.L., Wente S.R., O'Shea E.K. (2003). Regulation of chromatin remodeling by inositol polyphosphates. Science.

[23] Berger I., Fitzgerald D.J., Richmond T.J. (2004). Baculovirus expression system for heterologous multiprotein complexes. Nat. Biotechnol..

[24] Lowary P.T., Widom J. (1998). New DNA sequence rules for high-affinity binding to histone octamer and sequence-directed nucleosome positioning. J. Mol. Biol..

[25] Routh A., Sandin S., Rhodes D. (2008). Nucleosome repeat length and linker histone stoichiometry determine chromatin fiber structure. Proc. Natl. Acad. Sci. U.S.A..

[26] Norby J.G. (1988). Coupled assay of Na+, K+‐ATPase activity. Methods Enzymol..

[27] Jerabek-Williamsen M., Wienken C.J., Braun D., Baaske P., Duhr S. (2011). Molecular interaction studies using microscale thermophoresis. Assay Drug Dev. Technol..

[28] Linxweiler W., Hörz W. (1984). Reconstitution of mononucleosomes: characterization of distinct particles that differ in the position of the histone core. Nucleic Acids Res..

[29] Hamiche A., Sandaltzopoulos R., Gdula D.A., Wu C. (1999). ATP-dependent histone octamer sliding mediated by the chromatin remodeling complex NURF. Cell.

[30] Längst G., Bonte E.J., Corona D.F., Becker P.B. (1999). Nucleosome movement by CHRAC and ISWI without disruption or trans-displacement of the histone octamer. Cell.

[31] Luk E., Ranjan A., Fitzgerald P.C., Mizuguchi G., Huang Y., Wei D., Wu C. (2010). Stepwise histone replacement by SWR1 requires dual activation with histone H2A.Z and canonical nucleosome. Cell.

[32] Chen L., Conaway R.C., Conaway J.W. (2013). Multiple modes of regulation of the human Ino80 SNF2 ATPase by subunits of the INO80 chromatin-remodeling complex. Proc. Natl. Acad. Sci. U.S.A..

[33] Yao W., Beckwith S.L., Zheng T., Young T., Dinh V.T., Ranjan A., Morrison A.J. (2015). Assembly of the Arp5 subunit involved in Distinct INO80 Chromatin-Remodeling activities. J. Biol. Chem..

[34] Soultanas P., Dillingham M.S., Velankar S.S., Wigley D.B. (1999). DNA binding mediates conformational changes and metal ion coordination in the active site of PcrA helicase. J. Mol. Biol..

[35] Schubert H.L., Wittmeyer J., Kasten M.M., Hinata K., Rawling D.C., Héroux A., Cairns B.R., Hill C.P. (2013). Structure of an actin-related subcomplex of the SWI/SNF chromatin remodeler. Proc. Natl. Acad. Sci. U.S.A..

[36] Dominguez R. (2009). Actin filament nucleation and elongation factors—structure-function relationships. Crit. Rev. Biochem. Mol. Biol..

