# Supplementary material for: Synergy and antagonism in regulation of recombinant human INO80 chromatin remodeling complex
Source: Nucleic Acids Res. 2016 Jun 2;44(17):8179–88. doi: 10.1093/nar/gkw509 (PMC5041457; doi:10.1093/nar/gkw509)
Supplement: SUPPLEMENTARY DATA [file supp_44_17_8179__index.html]

Synergy and antagonism in regulation of recombinant human INO80 chromatin remodeling complex — Synergy and antagonism in regulation of recombinant human INO80 chromatin remodeling complex — Synergy and antagonism in regulation of recombinant human INO80 chromatin remodeling complex — SUPPLEMENTARY DATA 

# Synergy and antagonism in regulation of recombinant human INO80 chromatin remodeling complex

## SUPPLEMENTARY DATA

- SUPPLEMENTARY DATA
