# Supplementary material for: Synergy and antagonism in regulation of recombinant human INO80 chromatin remodeling complex
Source: Nucleic Acids Res. 2016 Jun 2;44(17):8179–88. doi: 10.1093/nar/gkw509 (PMC5041457; doi:10.1093/nar/gkw509)
Supplement: SUPPLEMENTARY DATA [file supp_gkw509_nar-02981-m-2015-File007.pdf]

Supplementary Figure Legends

Supplementary Figure 1 – Purified Ino80 core and SC2 complexes.

Supplementary Figure 2 – Raw data for MST measurements of Ino80 subunit deletion complexes.

Supplementary Figure 3 – Kinetic data for stimulated and unstimulated ATPase activity of Ino80 subunit deletion complexes.

Supplementary Figure 4 –ATPase activity in the presence of saturating H2A nucleosome.

Supplementary Figure 5 – Kinetic data for Ino80 ATPase activity over a range of ATP and IP_6 concentrations.

Supplementary Figure 6 – Kinetic data for Ino80 ATPase activity over a range of nucleosome and IP_6 concentrations.

Supplementary Figure 7 – Raw MST data of nucleosome biding in the presence of ligands. These experiments were conducted in the presence of 5 mM $MgCl_2$.

Supplementary Figure 1

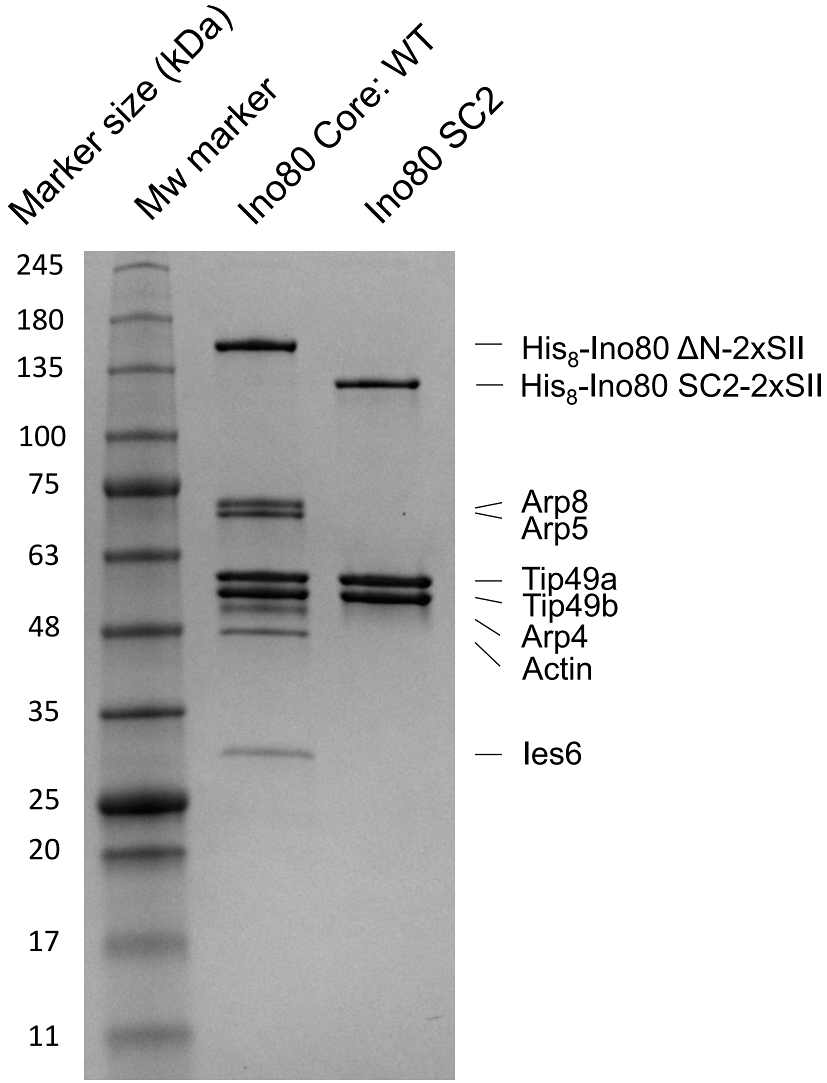

Supplementary Figure 2

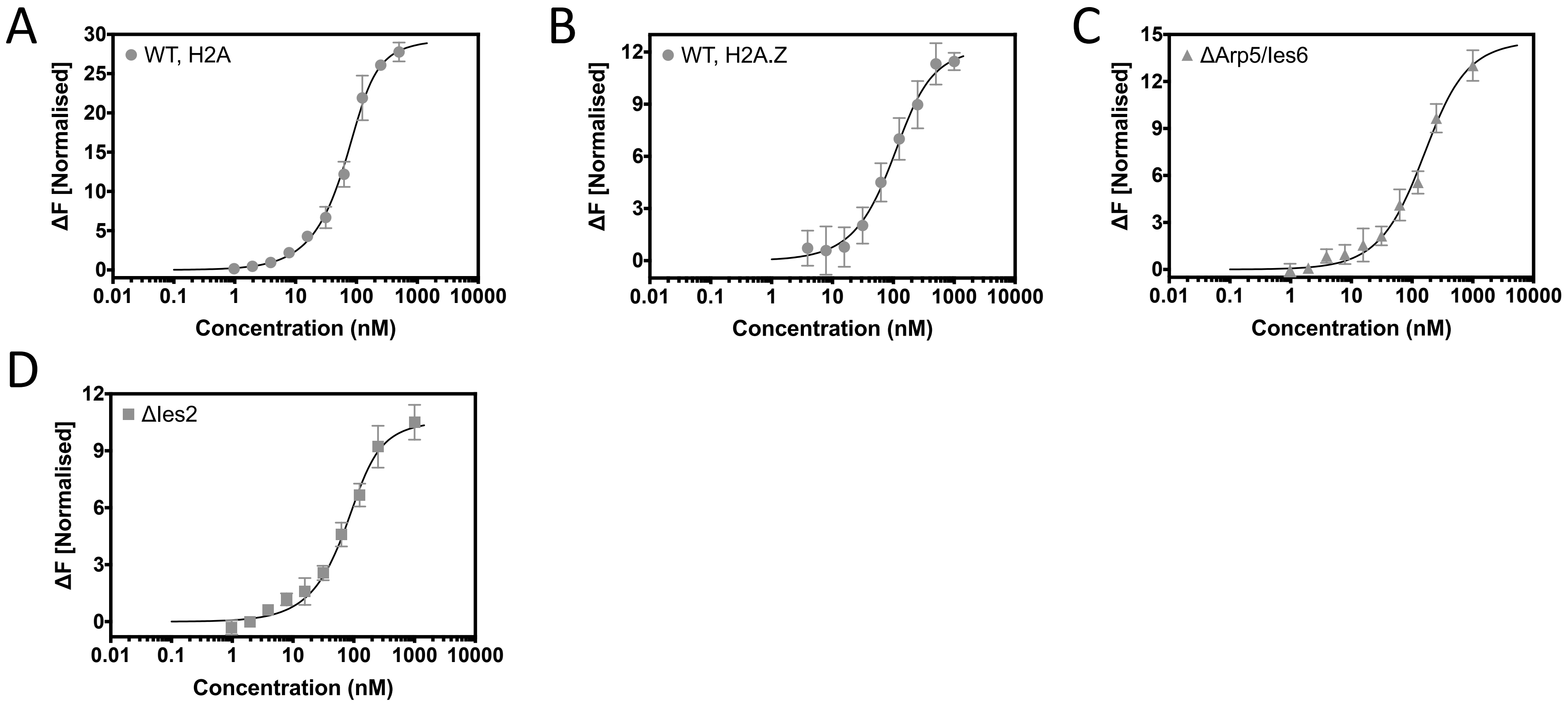

Supplementary Figure 3

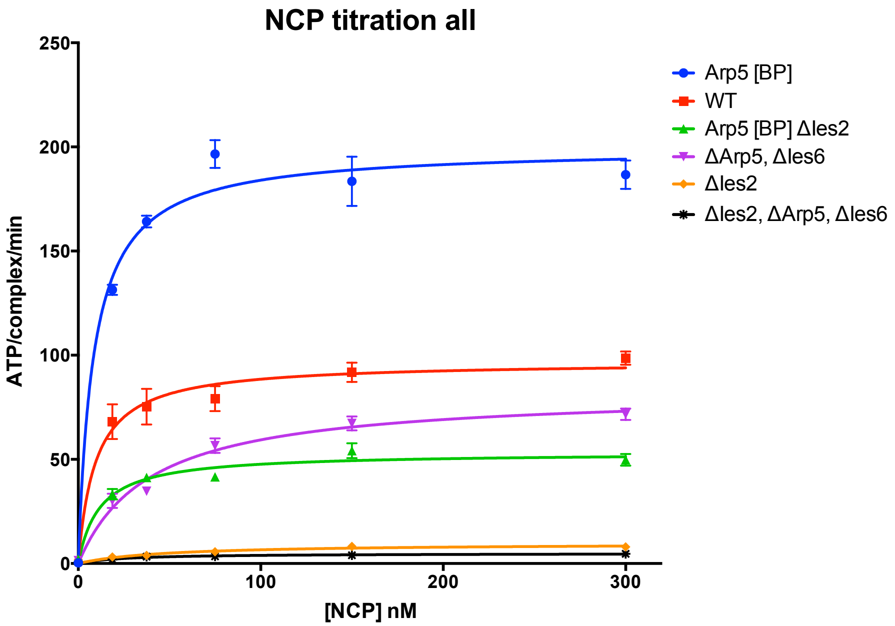

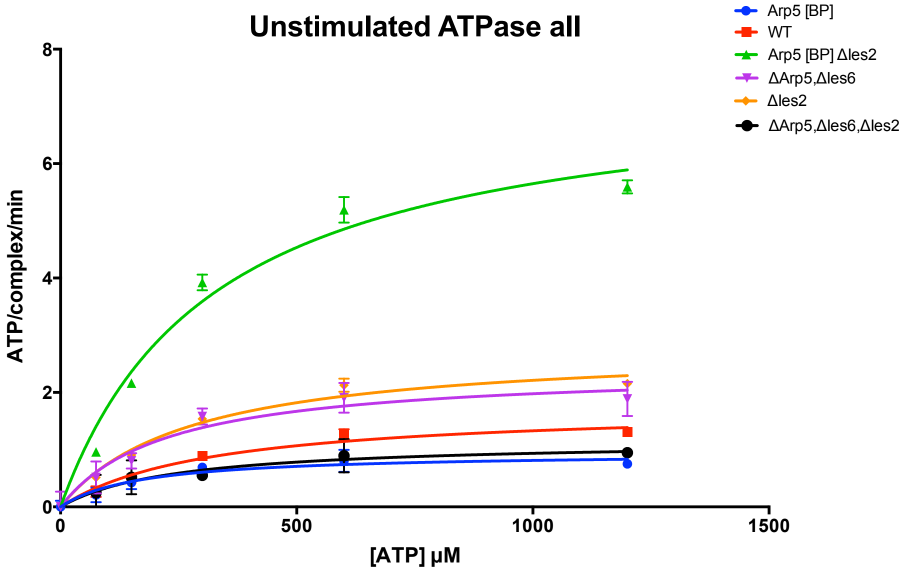

Supplementary Figure 4

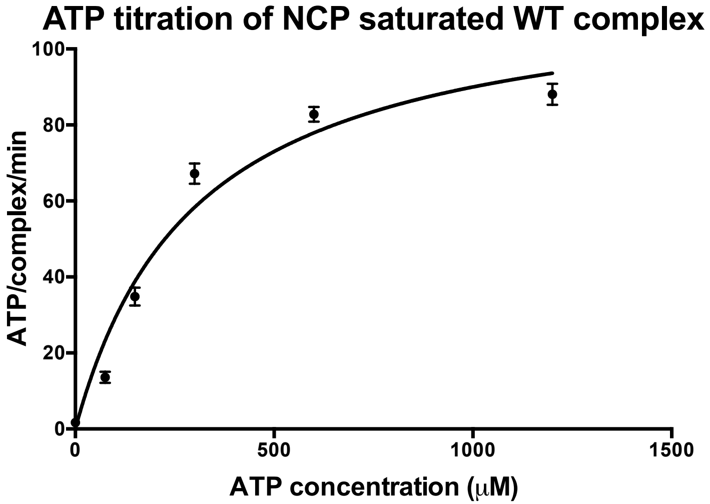

Supplementary Figure 5

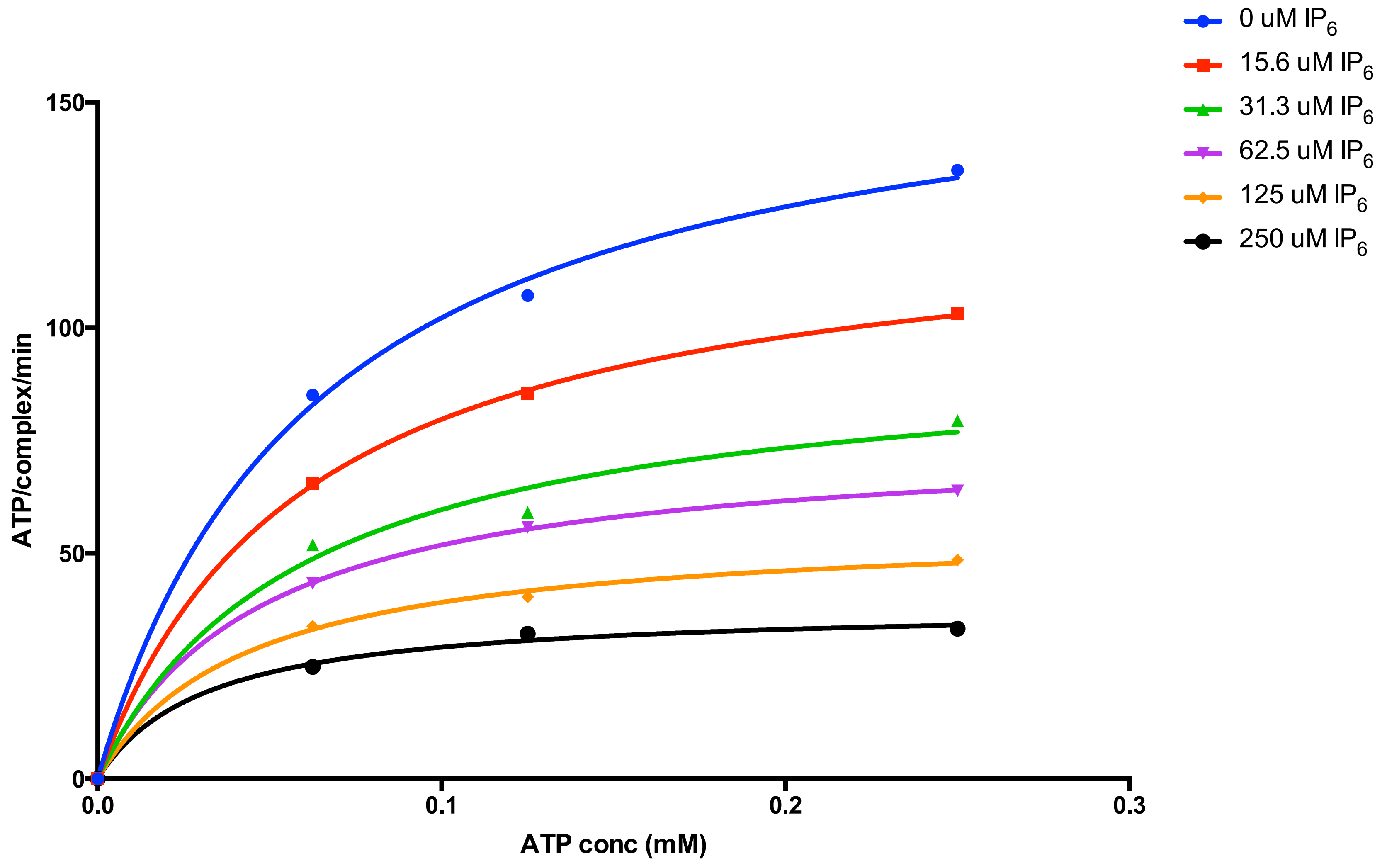

Supplementary Figure 6

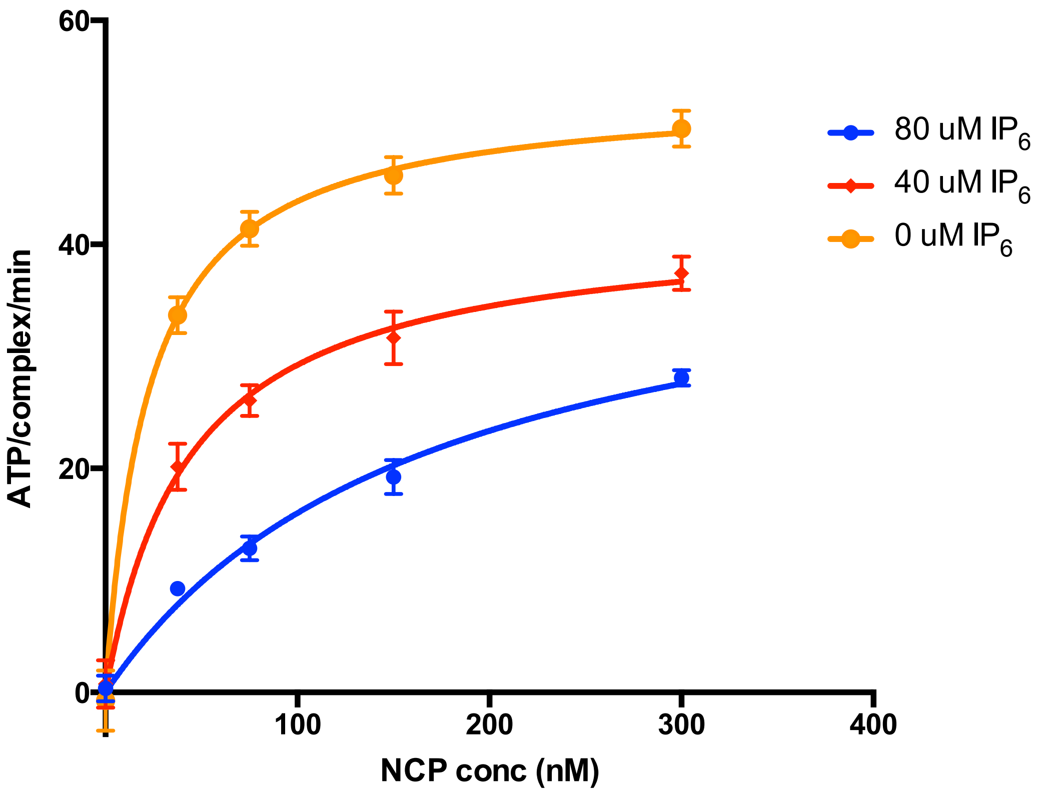

Supplementary Figure 7

Nucleosome = Human H2A-NCP, 100 bp overhang, **H3$^{\underline{AF647}}$**

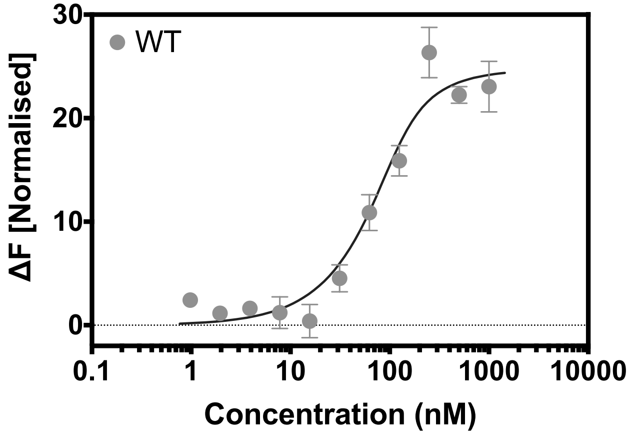

Kd = 22.9 ± 2.1 nM

Nucleosome = Human H2A-NCP, 100 bp overhang, **H3$^{\underline{AF647}}$**

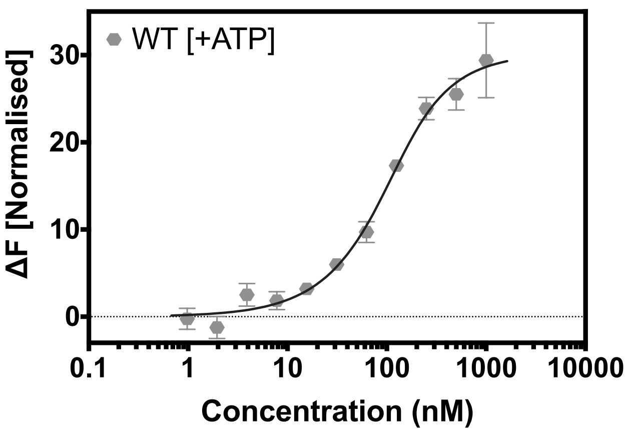

Kd = 53.3 ± 2.4 nM

Nucleosome = Human H2A-NCP, 100 bp overhang, **H3$^{\underline{AF647}}$**

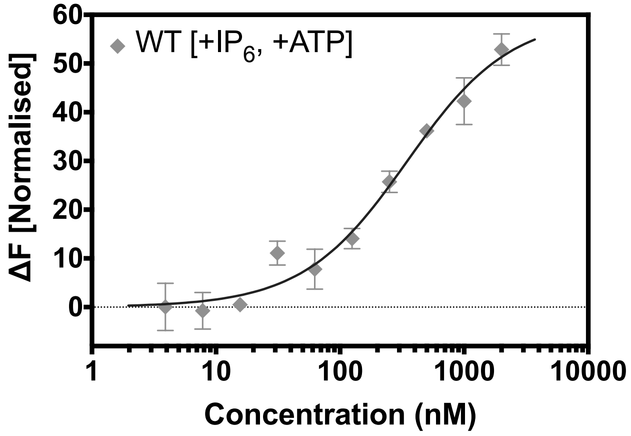

Kd = 321.0 ± 25.4 nM
